# Colexification Networks Encode Affective Meaning

**DOI:** 10.1007/s42761-021-00033-1

**Published:** 2021-05-15

**Authors:** Anna Di Natale, Max Pellert, David Garcia

**Affiliations:** 1 Center for Medical Statistics, Informatics and Intelligent Systems, Medical Univeristy of Vienna, Inffeldgasse 16c/I, Graz, 8010 Austria; 2 Institute of Interactive Systems and Data Science, Graz University of Technology, Inffeldgasse 16c/I, Graz, 8010 Austria; 3 Complexity Science Hub Vienna, Josefstaedterstrasse 39, Vienna, 1080 Austria

**Keywords:** Colexification, Semantics, Networks, Affective norms

## Abstract

Colexification is a linguistic phenomenon that occurs when multiple concepts are expressed in a language with the same word. Colexification patterns are frequently used to estimate the meaning similarity between words, but the hypothesis that these are related is still missing direct empirical validation at scale. Here, we show for the first time that words linked by colexification patterns capture similar affective meanings. Using pre-existing translation data, we extend colexification databases to cover much longer word lists. We achieve this with an unsupervised method of affective lexicon extension that uses colexification network data to interpolate the affective ratings of words that are not included in the original lexicon. We find positive correlations between network-based estimates and empirical affective ratings, which suggest that colexification networks contain information related to affective meanings. Finally, we compare our network method with state-of-the-art machine learning, trained on a large corpus, and show that our simple linguistics-informed unsupervised algorithm yields comparable performance with high explainability. These results show that it is possible to automatically expand affective norms lexica to cover exhaustive word lists when additional data are available, such as in colexification networks.

**Supplementary Information:**

The online version contains supplementary material available at 10.1007/s42761-021-00033-1.

## Introduction

Colexification is a linguistic phenomenon that occurs when a word can be used to express multiple concepts. Since its recent formalization (François, 2008), it has been the focus of extensive work that culminated in the creation of a dataset of cross-linguistic colexifications (List et al., 2018). This resource has been used to conduct studies in different areas, such as historical linguistics and language comparison. The second version of the dataset with enhanced language coverage (Rzymski et al., 2020) provides a more extensive resource that allowed the widening of its applications beyond the field of linguistics. In fact, the study of colexification allows the comparison of language structures across cultures and countries, contributing to a universal overview of the studied phenomena.

Colexification patterns have recently been analyzed to address the universality of emotion perception from a psychological perspective (Jackson et al., 2019). Jackson et al., (2019) show that understanding of emotions has a shared basis of universal meaning but differences exist in emotion interpretation across cultures. These differences can be partially explained by geographical distance. Moreover, considering some psychological dimensions of the selected concepts (three affective dimensions as well as approach-avoidance, certainty, and sociality), Jackson et al. show that valence and arousal are the two dimensions that perform best in differentiating clusters of emotional concepts. A similar approach has been used to study the human interpretation of a small subset of words referring to the celestial and landscape spheres (Youn et al., 2016). In that study, Youn et al., (2016) employed the occurrences of polysemies in different languages as a proxy for meaning similarity. Their analysis suggests that geographical and cultural differences have little significance in the human representation of the selected concepts, which share a universal understanding. This suggests that the structure of meanings in the human mind has common features, and in some cases there is little variance due to cultural and geographical distances.

Most studies that employ the concept of colexification rely on the assumption that colexification is linked to meaning similarity. This assumption is plausible from the definition of colexification and from results of previous studies, but the hypothesis of a relationship between colexification and meaning similarity is still to be directly tested at scale. A threat to the validity of this assumption is that colexification includes the phenomena of both homography and polysemy. A polysemous word has multiple meanings that are in some way related. For example, the word “crane” refers to both an animal and a construction machine, which are related because of their similar shape. Since polysemous words refer to multiple concepts, they are also instances of colexification. On the contrary, homographs are words that share the same written form but refer to multiple concepts that might not be semantically related. For example, “bear” can refer to an animal, while also being a verb meaning “to carry, to endure”; two meanings that have no relationship. Homographs are also instances of colexification but are devoid of meaning similarity. Homographs are a potential source of error in the use of colexification as an approximation to meaning similarity. An approach to mitigating this source of error is to consider several languages and language families when analyzing colexification patterns, but to date we have evidence of only the face validity of that approach. To frame previous research, we need an empirical test of the hypothesis that colexification is a good approximation for meaning similarity.

Our aim in this article is to test whether colexification patterns in multilingual resources are correlated with affective meaning similarity between words. To quantitatively compare affective meanings, we employ affective norms lexica that contain words and ratings in various dimensions of the affective states they express. In previous research, the most commonly used dimensions are *valence* and *arousal* (Russell, 1980). Valence refers to the degree of positivity or negativity expressed by a word, while arousal links to the level of activity of the emotions associated with a word. These concepts map to the dimensions of evaluation and activation in the analysis of questionnaires (Osgood et al., 1957), which can be expanded in turn with dimensions of dominance or potency and unpredictability (Fontaine et al., 2007). For the English language, two lexica are typically used in the literature: the WKB lexicon (Warriner et al., 2013), which includes ratings of valence, arousal, and dominance for nearly 14,000 English lemmas, and the more recent NRC VAD lexicon (Mohammad, 2018), which uses a *Best–Worst Scaling* scheme to derive crowdsourced ratings for more than 20,000 words. To account for uncertainties, multiple raters for each word are recruited on the online platforms, quantifying split-half reliability to assess agreement among raters.

In this article, we show that words that share colexification patterns tend to have similar affective meanings. We analyze three colexification networks: one based on a database of instances of colexifications and two based on identical translation databases. We present a simple algorithm to estimate the affective ratings of words based on their neighbors in a colexification network. We test this algorithm by calculating the correlation between the affective ratings of words and their estimates in a cross-validation exercise. We compare these correlations to the ones obtained by machine learning methods and illustrate how our algorithm can be used to expand affective norms lexica. More broadly, our findings demonstrate that affective science methods can be used to test fundamental properties of semantic meaning across databases of thousands of concepts.

## Methods

### Colexification Networks

*Colexification* is a linguistic phenomenon that occurs when two different concepts are expressed using the same word in one language. An example of colexification is the ancient Greek word “$\phi \acute \alpha \rho \mu \alpha \kappa \textit {o}\eta $” (“pharmacon”), which expresses the two concepts of “medicine” and “poison.” In this case, ancient Greek is said to *colexify* those two concepts, as they are expressed by the same word. As the example suggests, some colexification patterns are related to meaning similarity (François, 2008; Jackson et al., 2019), while others can arise because of geographical, historical, and cultural factors or because of coincidence, in the case of homography, for example.

It is important to note that the definition of colexification is based on the notion of concept. Since the definition of concept is labile, tracking colexifications in language is inherently complicated. To automatically detect colexification patterns in language and build a more extensive database, we use identical translations recorded in multilingual dictionaries and other digital resources. We define an *identical translation* as a situation in which two words in one language translate to the same word in a second language. Note that identical translation differs from colexification in the sense that colexifications link concepts, while identical translations link words. In the case of unambiguous and non-polysemous words, colexification and identical translation directly correspond to each other, while in cases of ambiguity, colexification patterns are more specific. For example, while colexifications distinguish between the two concepts “fly (insect)” and “fly (move through air),” identical translations take into account only the word “fly” for both meanings. The definition of identical translation constitutes a whole new computational approach to infer colexification patterns in an unsupervised and scalable fashion.

In this paper, we consider one colexification database, CLICS^3^, and two identical translation databases built from online resources: OmegaWiki, a collaborative project to produce a multilingual dictionary, and open-source bilingual dictionaries from FreeDict. CLICS^3^ (database of cross-linguistic colexifications) (Rzymski et al., 2020) is an expert-based database based on the definitions and classifications of concepts of Concepticon (List et al., 2020). It aggregates data from 30 different linguistic databases and is one of the most extensive linguistic resources with respect to language coverage. CLICS^3^ and its previous versions have been used to analyze colexification patterns in various fields beyond linguistics. Alongside CLICS^3^, we built two crowdsourced databases of identical translations from online resources: FreeDict and OmegaWiki.

FreeDict (freedict.org) is a website that collects several open-source, free bilingual dictionaries. We retrieved from the website the dictionaries that feature English either as translated or as translation language. This choice follows from the fact that our analysis is based on English word ratings. We collect and compare the translations of words in bilingual dictionaries that feature the same pair of languages as follows: First, we translate the English word *w* to the word *v* in another language. Second, we translate the word *v* back to English, resulting in word *u*. If the words *w* and *u* are different, we record an instance of identical translation between these two words in our database.

The second source of translation we consider is OmegaWiki. OmegaWiki was a collaborative project of the Wikipedia community to produce a free, multilingual dictionary for every language. To this end, users translate definitions given in English rather than words across languages. For example, they do not translate the single word “age” but one of its definitions as in “A period of history having some distinctive feature” or “To begin to look older; to get older.” This procedure makes it possible to distinguish senses of polysemous words and homographs and allows for a low error rate in the translations. From this resource, we download the translated words and organize them in a dictionary with English words and their translations. Every time a word in a language different than English is translated into two different English words, we have an instance of identical translation. Unfortunately, the original Web page for the OmegaWiki project is no longer online, but we share the data that we collected for this study in a GitHub repository (https://github.com/AnnaDiNatale/colex_affective).

**Fig. 1:**
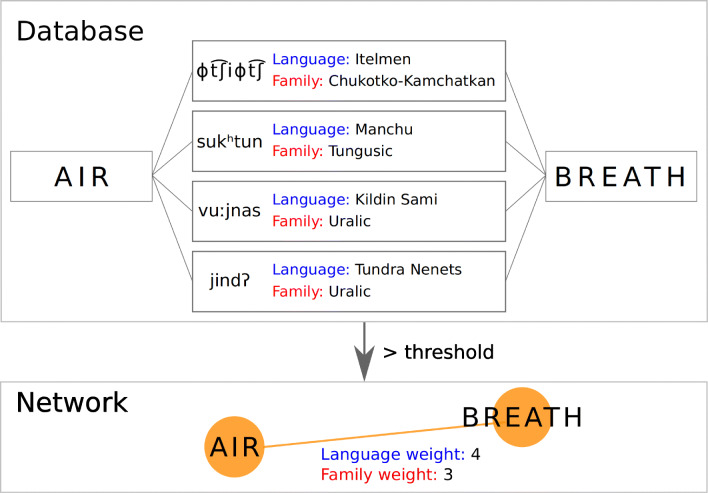
Construction of a colexification network. Nodes represent concepts (words in the case of identical translation) and links represent the existence of a colexification or identical translation pattern between the nodes it connects. Links are weighted by the number of languages and the number of families of languages that present the same connection. We usually apply a threshold on the weights of the links we consider

We construct a *colexification network* from each of our three databases. In the case of CLICS^3^, the nodes represent concepts and the links colexification patterns between the concepts represented by the nodes. In the colexification networks of FreeDict and OmegaWiki, nodes represent words and links correspond to identical translation occurrences between nodes. All these networks are undirected because the patterns we analyze are always invertible. As in previous work (List et al., 2013), the links of our colexification networks are weighted by the number of languages and the number of language families that present the same colexification or identical translation pattern (see Fig. 1).

We summarize descriptive statistics for our three networks in Table 1. Languages and language families are counted based on Glottolog (Nordhoff and Hammarström, 2011), a database of the world’s languages, language families, and dialects. Among our three databases, CLICS^3^ includes the most languages. It also features the shortest word list but, being to some extent manually curated, we can consider CLICS^3^ to have a low number of errors. The colexification networks built from FreeDict and OmegaWiki have much higher coverage of the English vocabulary. However, CLICS^3^ has been specially designed to distinguish senses of polysemous words, while the other two databases do not distinguish words in such cases and thus might lead to noisier networks.

**Table 1:** Descriptive statistics of the networks considered

Network	No. of languages	No. of families	No. of nodes	No. of links
CLICS^3^	2271	200	1647	4228
OmegaWiki	166	26	10,323	13,691
FreeDict	19	4	27,939	70,839

We report the number of languages, families of languages, nodes, and links featured in each network after cleaning

Colexification and identical translation patterns can arise for different reasons: meaning similarity, historical and geographical and cultural phenomena, as well as coincidence. To filter out cases in which their occurrences are not related to meaning similarity, we apply the same rule as in previous research using CLICS^3^ (Rzymski et al., 2020): colexifications that occur in less than 3 languages and 3 families are excluded. For the cases of OmegaWiki and FreeDict, since they contain many more words but have a smaller set of languages, we apply the same rule but include all identical translations that occur in at least 2 languages. In the case of CLICS^3^, we also consider a varying threshold on the number of languages and analyze how this filtering affects our results.

### Affective Meanings Quantified Through Affective Ratings

Affective meaning is a rich phenomenon and its empirical analysis requires a way to measure the affective meanings of words. Early works in the quantification of meaning identified three distinctive dimensions (Osgood et al., 1957) which, when applied to affective meanings, were mapped into the dimensions of valence, arousal, and dominance (Bradley & Lang, 1999). While additional dimensions can be added to represent affect (Fontaine et al., 2007), valence, arousal, and dominance serve as a common space for comparing results and providing an approximation to quantify affective meanings. These three dimensions are often empirically measured by averaging the ratings of several individuals when rating a single word in isolation. In this study, we use previous resources on affective ratings as a way to quantify the three affective dimensions of valence, arousal, and dominance. We use two reference lexica of affective ratings of English words: WKB (Warriner et al., 2013) and NRC VAD (Mohammad, 2018). We use the information in colexification networks to decode the affective meanings of words, i.e., we estimate the ratings of valence, arousal, and dominance of nodes in the network. It is important to note that the two dimensions of valence and dominance are colinear, i.e., in such affective lexica, dominance ratings can be partly explained by valence ratings alone. Valence and dominance have a correlation of 0.717 in WKB and of 0.488 in NRC VAD. For completeness, we consider all three dimensions in our study but also test if colexification networks explain dominance beyond its correlation with valence.

The WKB lexicon collects the affective ratings of nearly 14,000 English lemmas (Warriner et al., 2013), extending the older ANEW lexicon (Bradley & Lang, 1999). The lemmas of WKB have been rated on a scale from 1 to 9 by US residents recruited on Amazon Mechanical Turk. Around 3% of the participants declared to be native speakers of a language different from English and most of them had a college or Bachelor’s degree. The NRC VAD lexicon expands the WKB lexicon to a total of 20,000 English words. All words in NRC VAD were annotated by participants recruited on the crowdsourcing platform CrowdFlower. Each task featured 4-tuples of words from which the words with the highest and lowest affective property of the task (valence, arousal, or dominance) had to be chosen (Best-Worst Scaling scheme). Participants were English native speakers from all over the world.

The two affective lexica we use cover thousands of words, but also limit our analysis in certain aspects. First, both lexica have ratings for English words by English speakers. Although there are shared structures of affective meanings across cultures, they also show some variations (Jackson et al., 2019). In this study, we focus on the affective understanding of English native speakers, but further work is required to validate the soundness of our method for other languages. Second, both lexica only contain ratings at the level of words without disambiguation. In the case of numeric ratings as in WKB, such disagreement is quantified by the standard deviation of their ratings (Pollock, 2018). Some words in WKB have mean ratings close to the middle of a scale but have high standard deviation, which indicates that these words might be polysemous. Our analyses will keep track of the standard deviation of affective rating estimates to illustrate whether polysemy is also observable in colexification networks.

We map the words of the affective lexica to the colexification networks through a matching procedure tailored to the format of the data used for network construction. In the case of the OmegaWiki and the FreeDict networks, this matching is straightforward by just identifying exact uncased string matches. In the case of CLICS^3^, since nodes represent concepts rather than words, matching needs to clean the strings that identify concepts. In CLICS^3^, concepts are identified by labels, some of which contain punctuation or are constituted by more than a word, as for example the nodes representing the concepts “breath or breathe” and “wash (clothes).” We remove all text within parentheses, so concepts like “wash (oneself)” and “wash (clothes)” are both matched to the word “wash” in the WKB and NRC VAD lexica. Since both affective lexica do not distinguish different concepts expressed by the same word (i.e., polysemous and homographs are not disambiguated), we use the single rating provided by the lexicon. In the case of concepts defined by more than one word without punctuation, we match each word and compute the mean of their affective ratings. For example, the valence, arousal and dominance ratings of the concept “breath or breathe” is the mean of the respective ratings in the considered lexicon for the single words “breath” and “breathe.” In these cases, a majority of the words in the concept’s labels have the same lemma and thus have very similar affective ratings.

We manually inspected the matches resulting from both rules and checked that they did not include systematic errors that could be avoided. A comprehensive list of all these matches can be found in the Supplementary Material. Our manual inspection only found two anecdotal instances that we could consider a mismatch. These are the concept “kind (sort of)” and the verb “break” which are matched to words with a high valence. Note that this issue is present only for the CLICS^3^ network because it is based on disambiguated concepts. However, every study that features the lexica NRC VAD and WKB has limitations related to the disambiguation of words, since the problem of words with multiple meanings has not been taken into account during the rating procedure of the words in these lexica.

We use the term *matching words* to refer to the words that can be matched between a colexification network and an affective lexicon. Words in a colexification network that are not matched to any word in an affective lexicon are called *non-matching words*. Table 2 reports the number of words matched per network and lexicon. The smallest network, namely CLICS^3^, has a low coverage of the lexica but a high number of its words are matched to the affective lexica. For networks with more nodes, such as OmegaWiki and FreeDict, the coverage of the lexicon gets larger than in CLICS^3^, but at the cost of having a lower coverage of the network given the limited size of the affective norms lexica.

**Table 2:** Number of words in the affective lexica matched in the colexification networks and coverage

Colex. network	Affective lexicon	No. of matching words	% of network	% of lexicon
CLICS^3^	WKB	1263	77%	9%
	NRC VAD	1337	81%	7%
OmegaWiki	WKB	3872	38%	28%
	NRC VAD	4841	47%	24%
FreeDict	WKB	8707	31%	63%
	NRC VAD	11,718	42%	59%

The coverage of the affective lexicon and of the colexification network is also reported

**Fig. 2:**
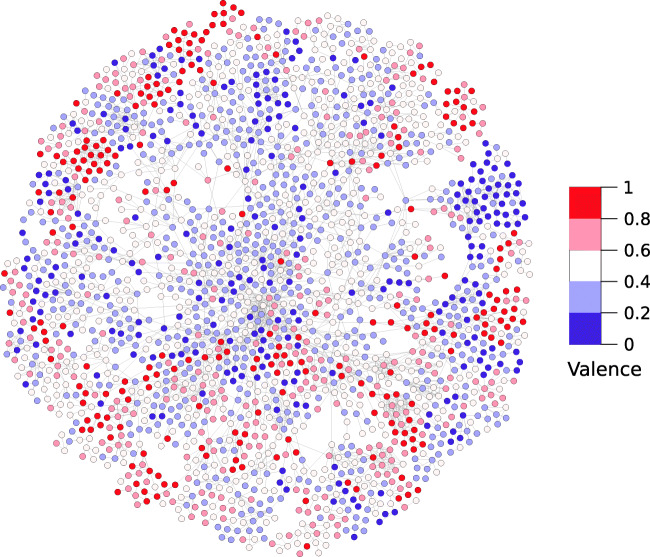
Network of matching nodes in OmegaWiki colored on the basis of their valence. The reference affective network is NRC VAD and the network is the largest connected component of the OmegaWiki network (42% of the matching nodes)

Figure 2 shows the subset of the OmegaWiki network composed of matching words in the NRC VAD lexicon. Nodes are colored according to the valence ratings in the NRC VAD lexicon, including only nodes in the largest connected component of the network. It can be noticed that nodes are clustered in small groups of similarly valenced words. The figure has been generated with Gephi (Bastian et al., 2009), using the Yifan Hu layout algorithm (Hu, 2005).

### Interpolating Affective Norms

**Fig. 3:**
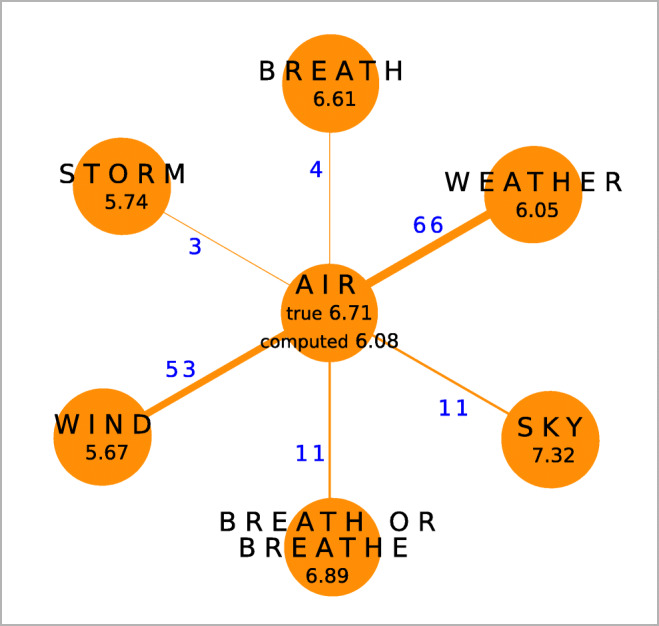
Computation of the weighted mean of the ratings of words in the networks. The valence rating of the word ‘air’ is computed given the valence ratings of its neighbors and the weight of the links

In order to test whether colexification networks encode affective similarity, we interpolate the ratings of a set of words (test set) on the basis of the true ratings of a training set of words. With *true ratings*, we refer to the ratings of the words in the affective lexicon. We estimate the valence, arousal, and dominance ratings of a word as the mean of the ratings of neighboring words in the colexification network, weighing the mean by the language weight of the links. Figure 3 illustrates this process: the valence of the word “air” is computed as the weighted mean of the values of its neighbors. We repeated this estimation using an unweighted mean as well as considering family weights, leading to very similar results that are reported in the Supplementary Materials. This indicates that the precise choice of the averaging rule does not play an important role.

**Fig. 4:**
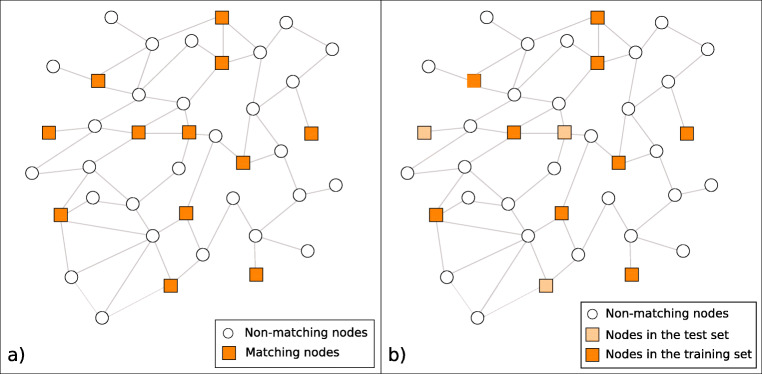
Graphical representation of the algorithms. Panel **a** represents the computation of the affective ratings of the non-matching nodes from the ratings of the words in the affective lexicon. Panel **b** represents the 75/25 split cross validation. The matching words are randomly split into training set and test set. The true ratings of the words in the training set are used to compute the ratings of all the other words. The performance is tested by computing the correlation between the true and estimated ratings for the words in the test set

The first test of the hypothesis that colexification networks track affective similarity is based on estimating valence, arousal, and dominance ratings of matching words. Matching words are also featured in the affective lexica, so they have true ratings of valence, arousal, and dominance with which to compare to our estimates. Our estimation runs iteratively on the network by computing affective values for non-matching words. We repeat this process until convergence is reached, i.e., the total change in estimates is below a predefined threshold. This way also nodes that do not have neighbors with true ratings but that can be reached through paths in the network will eventually have an estimate of affective ratings. After convergence, we estimate the valence, arousal, and dominance ratings of matching words as the language-weighted means of their neighbors. Figure 4a shows a graphical representation of the situation, where the squares represent the matching words and the circles represent the non-matching words. The non-matching words’ affective ratings are iteratively computed as estimates of the corresponding valence, arousal, and dominance, taken to be the weighted means of neighbors’ values. At convergence, the estimated ratings for matching words are computed as weighted means of the neighbors’ ratings, to compare later against the true values.

The three colexification networks are constituted by various connected components. If a connected component does not contain any matching word, it is not possible to compute the affective ratings of all the words in the component. In this case, all the nodes in the component are discarded. Therefore, usually, we are not able to compute the ratings for all the nodes in the network, and thus we track the coverage of each method to know how many words have ratings that cannot be estimated. Once each node in the network is assigned the computed ratings, we compare those ratings with the true ratings in the affective lexicon. We compute the correlation coefficient between the computed and the true ratings of this subset of words. Moreover, to understand how noise could be filtered in this process, we also compute the correlation coefficient considering only nodes that have a number of neighbors above a threshold. The computation of the affective ratings of words that have few neighbors could be strongly affected by error propagation. On the contrary, when a node has many neighbors, the signal can reach the node from multiple hubs, being therefore stronger and less influenced by noise.

In order to analyze the robustness of the method, we also study the role that rare colexifications play in preserving and transmitting the affective information in colexification networks. We repeat the computation described above, applying different language thresholds on the CLICS^3^ colexification network. We perform this analysis only on CLICS^3^ because the other colexification networks contain too few languages to allow this kind of analysis. The results are reported in the Supplementary Materials.

We compare our empirical results with the equivalent correlation coefficient in two *null models*. The first null model is a *random neighbors* model in which the matching nodes’ neighbors are chosen at random from the non-matching nodes, preserving the original degree distribution of the node. We then compute the ratings of those words, considering the original link weights. In the second null model, a *permuted values* model, the estimated ratings are permuted across the matching nodes, keeping the network structure unchanged. In both cases, we repeat the null model simulation 10,000 times and Fisher transform back and forth the mean of the resulting correlation coefficients.

We also analyze the influence of the colinearity of valence and dominance ratings on our estimates. We build two linear models, one for the true dominance ratings as a function of true valence ratings and one for true dominance ratings as a function of true valence ratings and the estimated dominance ratings. We then compare the residuals of the two models and compute the percentage of the residual variance of the true dominance ratings that is explained by the estimated dominance. This percentage indicates how much of the dominance ratings can be explained by the estimated dominance ratings on top of what the valence ratings already explain.

We further test the predictive power of our interpolation method for non-matching words and compare it with previous results using state-of-the-art machine learning methods. Previous works have provided methods to expand affective lexica using large text corpora by, for example, applying Latent Semantic Analysis to the ANEW lexicon (Bestgen & Vincze, 2012). For the task of inferring words’ affective ratings, the current state-of-the-art method applies word embedding techniques (Mikolov et al., 2013) to connect words to their nearest neighbors in a high-dimensional space. Since this approach clearly outperforms previous methods for the large word list of WKB, we use the results of Mandera et al., (2015) as the reference to frame our results.

Testing the quality of affective rating estimates from our interpolation method serves to assess whether colexification networks can be used to expand existing affective lexica in an unsupervised fashion. In particular, we perform a 75/25 split cross-validation over 10 iterations as in Mandera et al., (2015), randomly splitting the matching words, such that 75% of those belong to the training sample, and evaluating the quality of our estimates on the remaining 25% (the test sample; see Fig. 4b). Given the ratings of the words in the training sample, we again compute ratings for all other words in the network. We then consider the words in the test set for statistical analysis. As before, we compute the correlation between the computed and true values of the test set words that belong to a connected component with at least one word in the training set. We record the coverage values in each case, as for the full network analysis. We compare the results with those from machine learning methods in Mandera et al., (2015), and with 10,000 simulations of the two null models aforementioned. Due to computing time limitations, we perform the test against null models only for one random 75/25 split of the set.

## Results

**Fig. 5:**
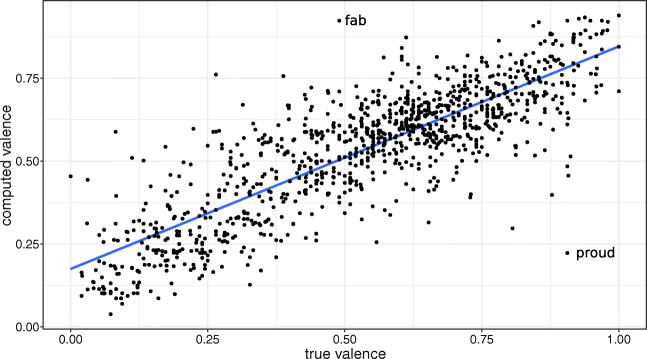
Valence ratings in the NRC VAD lexicon versus estimated ratings in the OmegaWiki network. The language weights are considered for the computation of the estimated ratings as weighted mean. Only nodes with at least 5 neighbors are plotted. The line shows a linear regression fit (*ρ* = 0.839, c.i. = [.82,.856], *p* < 10^− 5^). Labels indicate the most extreme outliers

We estimate the valence, arousal, and dominance ratings of words as the mean and weighted mean of the corresponding values of their neighbors in the colexification networks. We compute the correlation coefficient between the true ratings and the computed ratings for a specific set of words. In the first study, this set corresponds to the matching words. We also set a threshold on the number of neighbors of a node in order to include it in the analyzed set, and also analyze how this threshold affects the results. As an example of these correlations, Fig. 5 shows the scatter plot of valence estimates in the OmegaWiki network using the true valence ratings from the NRC VAD lexicon. The weighted mean is computed according to language weights for a neighbor threshold of 5. The resulting correlation coefficient is *ρ* = 0.839 (c.i. = [.82,.856], *p* < 10^− 5^).

**Fig. 6:**
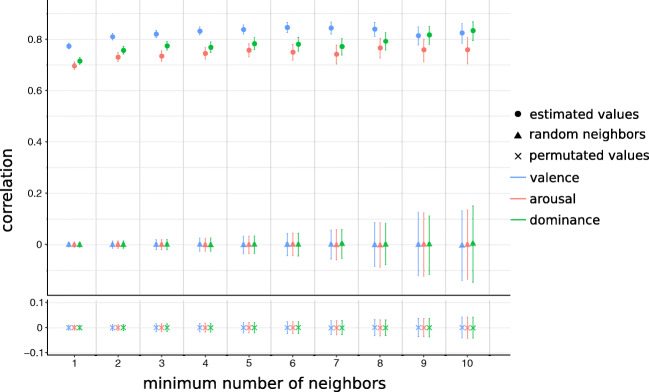
Comparison of the algorithm with the two null models. Here, the case of OmegaWiki with the NRC VAD lexicon is presented. The estimates using language-weighted means are reported. The bars represent 95% confidence intervals. The y axis represents the correlation between estimated and true ratings, while the x axis represents the number of neighbors the sample is filtered on. The higher this threshold is the fewer nodes are considered; therefore, the estimate has wider confidence intervals. The difference in correlation between the algorithm and the null models is significant with *p* values below 0.05

Similar results are obtained for different neighbor thresholds, even when considering all matching nodes regardless of the number of neighbors (i.e., a threshold of 0) (see Fig. 6). Table 3 reports the correlation coefficients between true and estimated values for all combinations of networks and affective lexica with a neighbor threshold of 0. Correlation coefficients are in the order of 0.7 for valence, arousal, and dominance in NRC VAD and between 0.48 and 0.75 for WKB. Results in the same range are found also when considering unweighted means and family-weighted means, showing the robustness of the method. Tables for these cases are given in the Supplementary Materials.

**Table 3:** Correlation of the true affective ratings with the computed ones

Network	Affective lexicon	No. of new words	Coverage	V	A	D
CLICS^3^	WKB	351	98%	.688	.481	.534
	NRC VAD	284	98.4%	.688	.638	.635
OmegaWiki	WKB	2700	63.7%	.728	.527	.646
	NRC VAD	2323	69.4%	.774	.698	.716
FreeDict	WKB	11,304	71.6%	.741	.575	.649
	NRC VAD	8756	73.3%	.794	.72	.749

The valence (V), arousal (A), and dominance (D) ratings are computed as weighted mean of the neighbors of each node. The weight considered is the language weight. The affective ratings are computed for all the nodes of the colexification network and the number of new words for which ratings are computed is reported. The percentage of coverage of the matching nodes is also reported. All correlation coefficients are significant (*p* < 0.001)

We also assess the role of rare colexification links in the correlation between true and estimated ratings. We find that correlation coefficients do not depend greatly on the minimum language weight used to filter the colexification network. Details are reported in the Supplementary Materials, showing that our method is robust to the presence of rare colexifications that might connect distant components of the network. This is also a consequence of the algorithm, which takes into account each link’s weight and thus dampens the role of these exceptions.

We compare these results with two null models. In the first, the neighbors of each node are chosen at random, while in the second the computed ratings are permuted. We compute the correlation coefficients of the estimates with the true ratings on 10,000 repetitions of the null models. Also in this case, we filter the nodes on the basis of the number of neighbors they have, as shown in Fig. 6 in the case of the OmegaWiki network and the NRC VAD affective lexicon. Correlation coefficients do not greatly depend on the choice of the neighbor threshold, approaching general values around 0.8 in the case of OmegaWiki and NRC VAD. Furthermore, in all the combinations of colexification networks and affective lexica, *p* values are clearly below 0.05 when comparing the empirical correlation coefficients with their distributions in the null models.

**Fig. 7:**
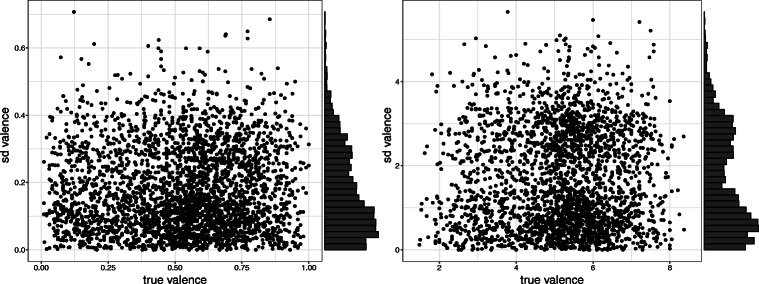
Standard deviation of the valence of nodes’ neighbors as function of their true valence ratings. The OmegaWiki network is considered for these plots. On the left, ratings in NRC VAD are represented while on the right WKB is considered. The bimodality of the standard deviations in WKB is observable and significant, while a dip test cannot reject the unimodality hypothesis of the distribution in NRC VAD

To understand the role of polysemous words through the rating schemes of the two affective lexica we use, we inspected the distribution of standard deviations of the ratings of neighbors of each node. Figure 7 shows the case of valence in OmegaWiki in both lexica, where we show the standard deviation of neighbor ratings versus the true valence of the node. One can see the absence of the characteristic shapes relating means and standard deviations for word ratings in WKB (Pollock, 2018). However, the distribution of standard deviations has a certain bimodal shape that can be seen as an indication of the presence of polysemous words, especially for the case of WKB ratings. We test the bimodality of the distribution of standard deviations with a dip test. We find that the distribution of standard deviation in WKB has a bimodal distribution with significant *p* value in all the affective dimensions in OmegaWiki and FreeDict and only for dominance in CLICS^3^. On the contrary, the same dip test does not permit the rejection of the unimodality hypothesis for NRC VAD except in the case of valence in CLICS^3^.

The two affective lexica that we consider present a high correlation between the dimensions of valence and dominance. This could lead to estimates of dominance ratings that do not add information to what valence can already predict. To test this, we compare two models: one that estimates dominance ratings as a linear function of valence ratings and a second one that considers both valence and the estimated dominance of method. This way we find that, in the case of NRC VAD, the estimated dominance can explain between 34 and 44% of the true dominance ratings not predicted by the true valence ratings. In the case of WKB, this value ranges from 6 to 12%. The values reported are significant with *p* value always below 10^− 3^ for all combinations of networks and lexica. This value is lower for WKB, probably because the correlation between valence and dominance is higher in that affective lexicon. The full table of results is reported in the Supplementary Materials.

Our algorithm to compute the affective ratings of the words in the colexification networks has some limitations. In particular, we use the affective ratings of a node to compute its neighbors’ ratings. In the case of a matching node, from its neighbors’ ratings, we recompute the values of the initial node. This way, a node’s true ratings are used to compute its estimated ratings. In order to overcome this problem, we perform a cross-validation analysis. To be able to compare to Mandera et al., (2015), we perform a 75/25 split cross-validation on 10 repetitions, similar to the method used for machine learning model evaluation. In some cases, it is not possible to compute ratings for all words because random splits might disconnect nodes from the nodes with ratings. Nonetheless, the number of those disconnected words is moderate, as it is always possible to compute more than 94*%* of the words in the test set (see Table 4). This coverage is higher than what can be achieved with corpus-based machine learning methods, which can estimate about 90% of word ratings (Mandera et al., 2015). Table 4 reports the correlation of true and estimated affective ratings in the 75/25 split cross-validation analysis. The performance of our methods is comparable to the one of Mandera et al., (2015) (95% CI overlap), but all our methods have higher coverage when compared with the WKB estimates of Mandera et al., (2015). The above results are similar when using unweighted means and family-weighted means to estimate word ratings. These results are reported in the Supplementary Materials.

**Table 4:** Results of the 75/25 split cross validation in comparison to Mandera et al., (2015)

Network	Affective lexicon	% computed words	V	A	D
CLICS^3^	WKB	99.1%	.663	.431	.524
	NRC VAD	99.3%	.649	.619	.607
OmegaWiki	WKB	94.5%	.653	.422	.557
	NRC VAD	94.6%	.728	.643	.661
FreeDict	WKB	98.7%	.668	.467	.561
	NRC VAD	98.6%	.747	.666	.700
Mandera et al., 2015	WKB	90.1%	.694	.478	.595

The affective ratings of a test set of words are computed as weighted mean of the ratings of their neighbors in the network. Each model is run 10 times and the means of the correlation coefficient is reported after applying Fisher Z transformation. Coverage over the test set is also reported. All correlation coefficients have *p* < 0.001

**Fig. 8:**
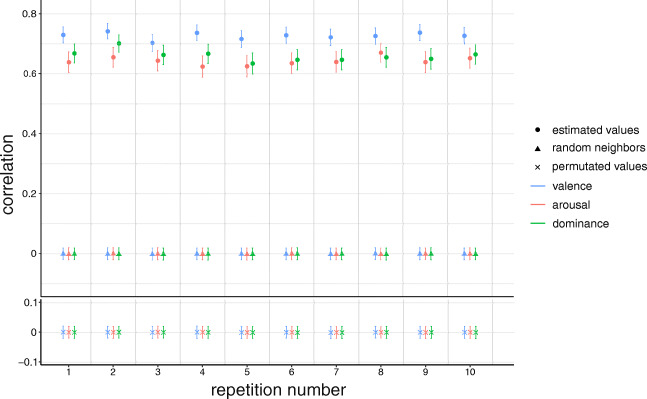
Outcomes of the 75/25 cross-validation in comparison with 10,000 repetitions of the two null models. 10 different splits of the matching words in the OmegaWiki network and the NRC VAD lexicon are considered. Bars show 95% confidence intervals. Means and confidence intervals of the null models are plotted after back-and-forth Fisher Z transformation

We compare the results of the 75/25 split cross-validation to the resulting correlations in 10,000 simulations of each null model for one of the 75/25 splits. The correlation coefficients of each estimate with the true rating are significantly higher (95% level) than the corresponding correlation in each null model. Figure 8 illustrates this result for 10 repetitions of the null models in the case of OmegaWiki and the NRC VAD lexicon. In each repetition, the correlations of our estimates and the true valence, arousal, and dominance are much higher than the resulting ones in each null model, which for both null models are concentrated around zero. This shows that the quality of the estimators based on colexification networks is not an artifact of some network structure particularity or inhomogeneity of the rating distributions.

All of the above results are similar when using unweighted means and family-weighted means to estimate word ratings. These cases are reported in the Supplementary Materials. Codes, data, and word lists to reproduce all these results are openly accessible at https://github.com/AnnaDiNatale/colex_affective.

## Discussion

We considered three colexification networks and interpolated the ratings of valence, arousal, and dominance of their words in two affective lexica. We evaluated this estimation in two exercises, one using the full network and another applying a cross-validation design to test the quality of these interpolations as predictors of affective ratings. In both cases, with significant and high correlation coefficients, we find that our colexification-based method is a strong predictor of the affective ratings of words in the network and that their results are comparable to the ones of machine learning approaches. Furthermore, comparing the results with two null models, we find that our estimates are not artifacts of particular network properties.

Some further insights can be noticed when considering outliers highlighted in Fig. 5. The most extreme outliers for valence in the case of the OmegaWiki network and the NRC VAD lexicon are the words “fab” and “proud.” The adjective “proud” has an estimated valence value of 0.223, much lower than its clearly positive rating in the NRC VAD lexicon (0.906). This happens because its neighbors in the network contain negative words such as “presumptuous,” “pretentious,” and “arrogant.” While all these words are similar in meaning to “proud,” English-speaking raters interpreted the affective meaning of “proud” as positive. We see this as an example of a cultural norm manifesting in a particular word, which might not be visible when studying affective norms in other languages. The positive rating of such a word can be seen as resulting from the limitations of affective lexicon creation. In fact, nearly all the participants were English native speakers. We conjecture that, if we only use English and related languages, the correlation between estimated and true affective values will be stronger, but with a lower coverage.

Moreover, in the scale of NRC VAD, the noun “fab” has a neutral rating with valence value of 0.49. The OmegaWiki colexification network estimates the valence of “fab” to be 0.923. The neighbors of “fab” in the network are “fabulous,” “beautiful,” “great,” “glorious,” “marvellous,” “astonishing,” and “wonderful,” which explain this high positive valence estimate. As “fab” is a slang abbreviation of “fabulous,” its strong positive meaning is evident, pointing to an issue of its rating in NRC VAD. Our interpretation of this discrepancy lies in the method used to estimate affective values in NRC VAD. The Best-Worst scaling method of NRC VAD uses comparisons of four words to generate a single scale, such that raters select the most positive and most negative word of the four (Mohammad, 2018). This enables perfect comparison of five of the pairs between the four words, but does not allow comparison between the two words in the middle. If raters avoid selecting words that they do not understand as the most positive or the most negative, a low level of familiarity with the word “fab” explains why it lies close to the center of the scale. This pattern could be used to improve affective ratings in existing lexica, at least by identifying systematic errors through examples like this one.

Our work builds on Osgood’s seminal works that developed the method of the semantic differential to measure meaning similarities. Osgood’s semantic spaces are constructed by the neighboring words sharing a similar pattern of coordinates on a scale of selected polar adjectives with a “meaningless” origin point. The redundancy of selected adjective pairs is then revealed through factor analysis to yield the classic three dimensions of evaluation, potency, and activation (Osgood et al., 1957). We propose the different criterion of colexification to construct networks that do not depend on well-argued but ultimately hand-crafted sets of adjectives as in the semantic differential. Like Osgood, we learn from the close inspection of outliers (like “fab” and “proud”): Osgood’s exploration of cross-cultural constants finds that “progress” was linked to much lower “activation” (arousal) than elsewhere in the world in a sample of Mexican Yucatans. It turned out that “Progreso” is the name of a seaside resort town that is famous for relaxing on the beach (Osgood, 1971).

Our method to estimate valence, arousal, and dominance values showed higher correlations when employing ratings from the NRC VAD lexicon than when using the WKB lexicon. These higher correlation coefficients with NRC VAD should not be seen as outperforming Mandera et al., (2015), since that article only used WKB for evaluation. The high performance with NRC VAD can be attributed to the higher reliability score of its annotations when compared to the WKB lexicon. It is worth noting that the upper limit for the performance of estimators when compared to word ratings is not 1 due to the bounded reliability of affective lexica. In particular, WKB has a split-half reliability of 0.914 for valence, 0.689 for arousal, and 0.77 for dominance while NRC VAD reports a split-half reliability of 0.95 for valence, 0.90 for arousal, and 0.91 for dominance.

The results presented here are robust to different ways of calculating means over the neighborhood of a word. In particular, they are very similar when using unweighted means, family weights, and language weights. Furthermore, we calculated correlations over various thresholds of the minimum number of neighbors and the minimum incidence of a colexification pattern to estimate the ratings of a word. We find high correlations even when using no threshold and the results are consistent for threshold values up to 10. This illustrates the robustness of colexification networks for estimating affective ratings of words.

By comparing results between colexification networks generated with a colexification database (CLICS^3^) and networks generated from identical translation databases (OmegaWiki and FreeDict), we provide a first test of the validity of crowdsourced colexification networks. Future research can test the validity of these methods based on identical translation when analyzing other ways of capturing word meanings or similarity between pairs of words. Our findings suggest that affect undergirds meaning structures, calling for further research in how affective ratings predict semantic relationships between concepts or words. Although our method is a simple, unsupervised computation, its accuracy is comparable to machine learning methods and it shows higher coverage over words in lexica of affective ratings. Our results can be considered as a step towards macrolinguistics, and a contribution to methods for automatically expanding and improving affective norms lexica. In particular, our methods allow the expansion of existing lexica to include more words. For example, our method can add more than 11,000 words to the WKB lexicon. Further research can explore more advanced techniques that combine our theory-inspired method with machine learning approaches to set a higher standard for affective rating interpolation.

## Conclusions

In this paper, we tested whether colexification patterns are correlated with affective meanings by showing how colexification networks can be used to estimate the ratings of valence, arousal, and dominance of words. Although the method is based on a simple algorithm, we prove that its accuracy is comparable to that of state-of-the-art techniques, in particular, to machine learning approaches. This practice has the potential to lower the costs of lexica creation, which usually requires a study to be designed, and a group of nonexpert participants to be recruited. Closely analyzing words for which the algorithm fails to infer the affective rating in established lexica shows that this method might possibly improve the practice of word ratings. In fact, the method might recognize systematic errors in the lexicon, calling for additional ratings that can fix these problems.

This article presents a combination of approaches from linguistics, psychology, and computer science that yield new insights into an affective science question. Using three different colexification networks, two computationally estimated from identical translation data, we showed that noisy data, when of large scale and including structural information, can be used to accurately expand affective norms lexica and empirically test a frequent assumption of linguistics research.

## Electronic supplementary material

Below is the link to the electronic supplementary material.

(PDF 1.56 MB)

## Additional Information

### **Data Availability**

The datasets generated and analyzed during the current study are available at https://github.com/AnnaDiNatale/colex_affective

### **Code Availability**

All codes are available at https://github.com/AnnaDiNatale/colex_affective
