# Supplementary material for: Colexification Networks Encode Affective Meaning
Source: Affect Sci. 2021 May 15;2(2):99–111. doi: 10.1007/s42761-021-00033-1 (PMC9382918; doi:10.1007/s42761-021-00033-1)
Supplement: Supplementary file 1 — (PDF 1.56 MB) [file 42761_2021_33_MOESM1_ESM.pdf]

Supplementary materials for 'Colexification networks encode affective meaning'

Anna Di Natale*[,1,2,3], Max Pellert[1,2,3], and David Garcia[2,1,3]

[1]Medical University of Vienna
[2]Graz University of Technology
[3]Complexity Science Hub Vienna
*corresponding author, email: dinatale@csh.ac.at

Correlation of computed and true affective ratings

The Tables of the correlation coefficients of computed and true affective ratings are reported. The ratings are computed as unweighted and weighted means of the respective ratings of the neighboring nodes. Weighted means are computed considering family weights. In all cases, similar results are obtained.

Network	Affective lexicon	V	A	D
CLICS^3	WKB	.690	.488	.534
	NRC VAD	.690	.640	.640
OmegaWiki	WKB	.729	.528	.647
	NRC VAD	.775	.698	.717
FreeDict	WKB	.744	.579	.653
	NRC VAD	.795	.722	.751

Table 1: Correlation of the true valence (V), arousal (A) and dominance (D) ratings with the computed ratings as weighted mean of the ratings of the neighbors of each node. For the reported results, the family weights are considered. All correlation coefficients have $p < 0.001$

Network	Affective lexicon	V	A	D
CLICS^3	WKB	.686	.479	.532
	NRC VAD	.682	.622	.638
OmegaWiki	WKB	.731	.529	.65
	NRC VAD	.776	.698	.718
FreeDict	WKB	.745	.58	.656
	NRC VAD	.796	.724	.752

Table 2: Table of the correlation of the true valence (V), arousal (A) and dominance (D) ratings with the computed ratings as unweighted mean of the ratings of the neighbors of each node. All correlation coefficients have $p < 0.001$

Analysis of the role of rare colexifications

We consider the CLICS^3 network and filter it on the basis of the language weight of its links. We perform the analysis described previously with every version of the filtered network. We then compare the correlation coefficients obtained as a function of the link threshold. Figure 1 shows this comparison with WKB as ground truth for the affective ratings and 2 shows it for NRC VAD. In both cases, the correlation coefficients increase steadily in correspondence to higher values of the threshold. Nonetheless, the results show robustness with respect to the rareness of the links in the network.

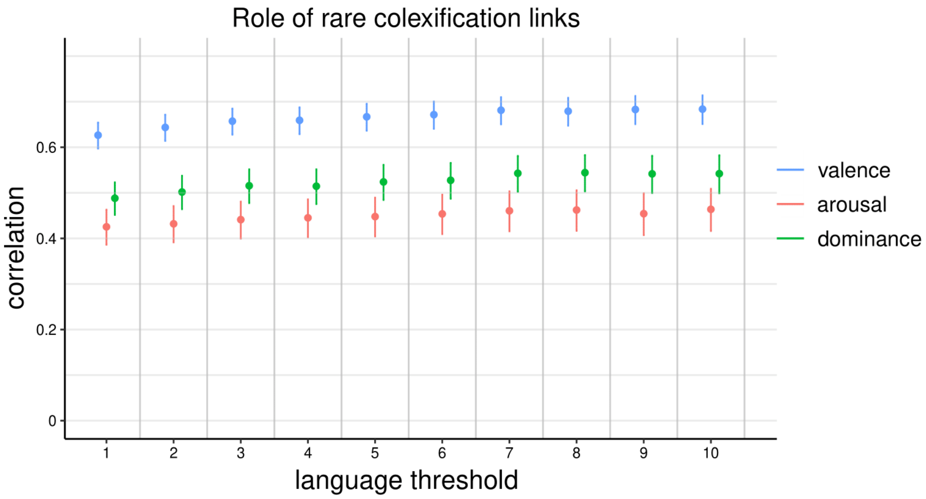

Figure 1: Role of rare colexification patterns on the correlation coefficients with ground truth values from WKB. We impose a threshold on the number of languages a link in the network CLICS^3 has to have in order to be considered for the computation. The values of the correlation steadily increase as function of the threshold.

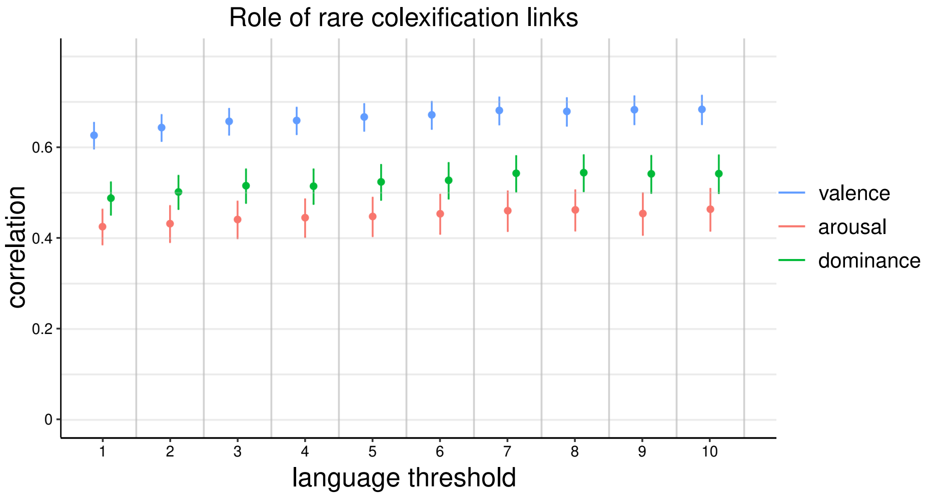

Figure 2: Role of rare colexification patterns on the correlation coefficients with ground truth values from NRC VAD. We impose a threshold on the number of languages a link in the network CLICS^3 has to have in order to be considered for the computation.

Standard deviation of words' neighbors

We consider the standard deviation of ratings of the neighbors of words in the colexification networks as function of the true valence of the word. The plots for valence in CLICS^3 (Figure 3) and FreeDict (Figure 4) are here reported. The results do not vary significantly when taking into account the other affective dimensions.

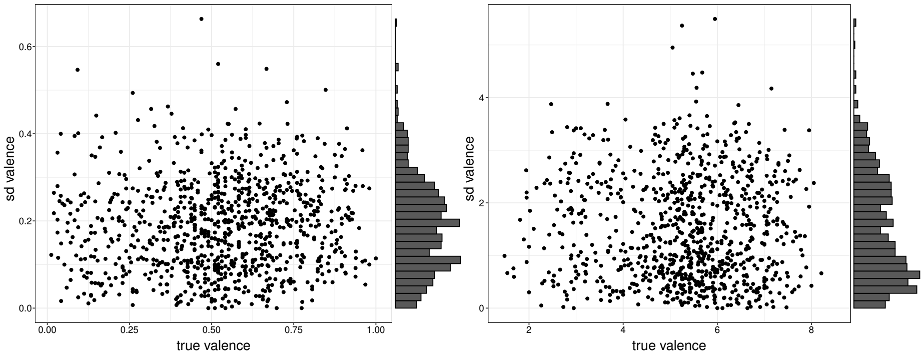

Figure 3: **Standard deviation of the valence of nodes' neighbors as function of their true valence rating.** The CLICS^3 network is considered for these plots. On the left, ratings in NRC VAD are represented while on the right WKB is considered

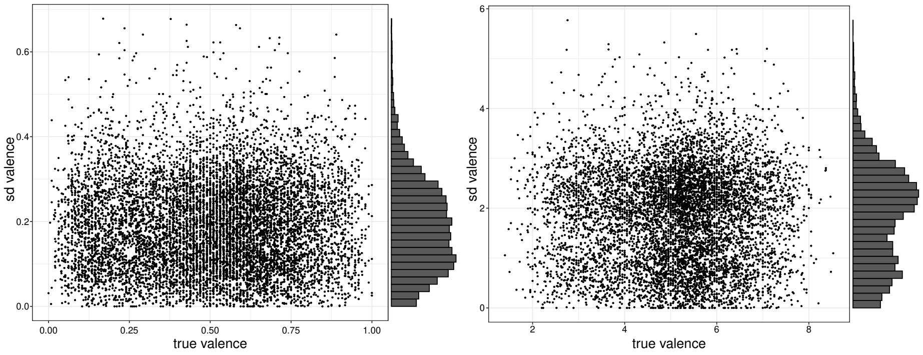

Figure 4: **Standard deviation of the valence of nodes' neighbors as function of their true valence rating.** The FreeDict network is considered for these plots. On the left, ratings in NRC VAD are represented while on the right WKB is considered

Colinearity analysis

We analyse the role on our study of the colinearity between the valence and dominance ratings. We compare the residuals of two linear models to determine the influence of colinearity between valence and dominance on our estimates. We find that on top of the predictive power of valence, our estimations of dominance ratings can explain up to 44% of the true Dominance ratings. Table 3 reports the results of such analysis, which are significant with p-value always smaller than 10^{-3}.

Network	Affective lexicon	share
CLICS^3	WKB	6.7%
	NRC VAD	34.5%
OmegaWiki	WKB	12.6%
	NRC VAD	40.6%
FreeDict	WKB	12.6%
	NRC VAD	44.0%

Table 3: **Share of the true dominance ratings that is explained by the estimated dominance on top of the estimates of valence alone.** The differences between WKB and NRC VAD are probably due to the higher colinearity value in WKB

75/25 split cross validation

The Tables that follow summarise the results for the 75/25 split cross validation test when considering unweighted means and means weighted on the family weights.

Word network	Affective lexicon	V	A	D
CLICS^3	WKB	.662	.420	.523
	NRC VAD	.644	.600	.615
OmegaWiki	WKB	.655	.421	.560
	NRC VAD	.730	.641	.662
FreeDict	WKB	.669	.466	.565
	NRC VAD	.748	.666	.701

Table 4: Results of the 75/25 split cross validation. Each algorithm is run 10 times and the means of the correlations are reported. In this case, the method deploys unweighted means. All correlation coefficients have $p < 0.001$

Network	Affective lexicon	V	A	D
CLICS^3	WKB	.668	.439	.527
	NRC VAD	.654	.624	.618
OmegaWiki	WKB	.653	.423	.556
	NRC VAD	.729	.642	.662
FreeDict	WKB	.669	.467	.562
	NRC VAD	.748	.666	.701

Table 5: Results of the 75/25 split cross validation. Each model is run 10 times and the means of the correlations are reported. In this case, the model computes means weighted on the number of families. All correlation coefficients have $p < 0.001$
